# Supplementary material for: Electrodynamics of photonic temporal interfaces
Source: Light Sci Appl. 2025 Sep 23;14:338. doi: 10.1038/s41377-025-01947-2 (PMC12454649; doi:10.1038/s41377-025-01947-2)
Supplement: Supplementary file 1 — Supplementary material [file 41377_2025_1947_MOESM1_ESM.docx]

**Supplementary Material for**

**Electrodynamics of Photonic Temporal Interfaces**

Emanuele Galiffi^1,*^, Diego Martinez Solís^2^, Shixiong Yin^1,3^, Nader Engheta^4^ and Andrea Alù^1,3,5,*^

^1^ Photonics Initiative, Advanced Science Research Center, City University of New York, New York, USA

^2^ Departamento de Tecnología de los Computadores y de las Comunicaciones, University of Extremadura, 10003 Cáceres, Spain

^3^ Department of Electrical Engineering, City College of The City University of New York, New York, NY, 10031 USA

^4^ Department of Electrical and Systems Engineering, University of Pennsylvania, Philadelphia, PA, 19104, USA

^5^ Physics Program, Graduate Center of the City University of New York, New York, NY, 10016, USA2

*egaliffi@gc.cuny.edu

*aalu@gc.cuny.edu

1. **Numerical field plots for the non-dispersive scenarios**

The analytic results to the plethora of perfectly abrupt temporal interfaces shown in the main text can be also retrieved numerically, with error-controllable precision, by first approximating the Heaviside step function with some smooth $C^{1}$-continuous—though rapidly changing—function, e.g.

$$\theta(t)\approx\frac{1+\tanh(\alpha t)}{2}$$

with parameter $\alpha$ parameterizing the velocity of the temporal transition. This approximation will help us induce the temporal discontinuities in the numerical model of the polarization hereinafter.

Assuming, initially, unbounded plane-wave propagation of the form $e^{i\left( \omega t-kz \right)}\hat{x}$, and considering momentum ($k$) conservation, we can work with $k$-space phasor $E_{x}\left( t \right)$ such that:

$$\tilde{E}_{x}(t,z)=E_{x}(t)e^{(-ikz)}$$

In the absence of magnetic response, the curl equations are thus reduced to a pair of ordinary differential equations (ODEs):

which can be merged and made second order in the form of the wave equation, or else straightforwardly solved in a staggered finite-difference time-marching scheme [1].

All that remains is the modeling of the specific microscopics of the polarization response in Eq. . Assuming no dispersion, we here identify two distinct scenarios for the instantaneous dielectric response $D_{x}=\varepsilon E_{x}$ when $\varepsilon$ becomes time-varying:

$$ikH_{y}(t)=\partial_{t}[\varepsilon(t)E_{x}(t)]$$

conserves total charge (continuous $D$) and describes the dynamics depicted in Figs. 2a-b and 2c-d in the main manuscript, whereas:

$$ikH_{y}(t)=\varepsilon(t)\partial_{t}E_{x}(t)$$

leads to continuous $E_{x}$ across the discontinuity in $\varepsilon(t)$ and dictates the physics of Figs. 2e-f and 2g-h. This discontinuity is parameterized in both cases as:

$$\varepsilon(t)=\varepsilon_{1}+(\varepsilon_{2}-\varepsilon_{1})\theta(t)$$

The resulting numerical field plots calculated with our in-house finite-difference time-domain code for the four non-dispersive time-interfaces considered are shown in Fig. S1. Note how the continuity and discontinuity of the respective displacement and electric fields is also manifested in the appropriate time-domain simulations, and how the magnetic field exhibits a discontinuity in its first derivative in the cases (a) and (b), whereby the electric field is discontinuous, as expected from Faraday’s law, whereas it is continuous for (c) and (d).

**
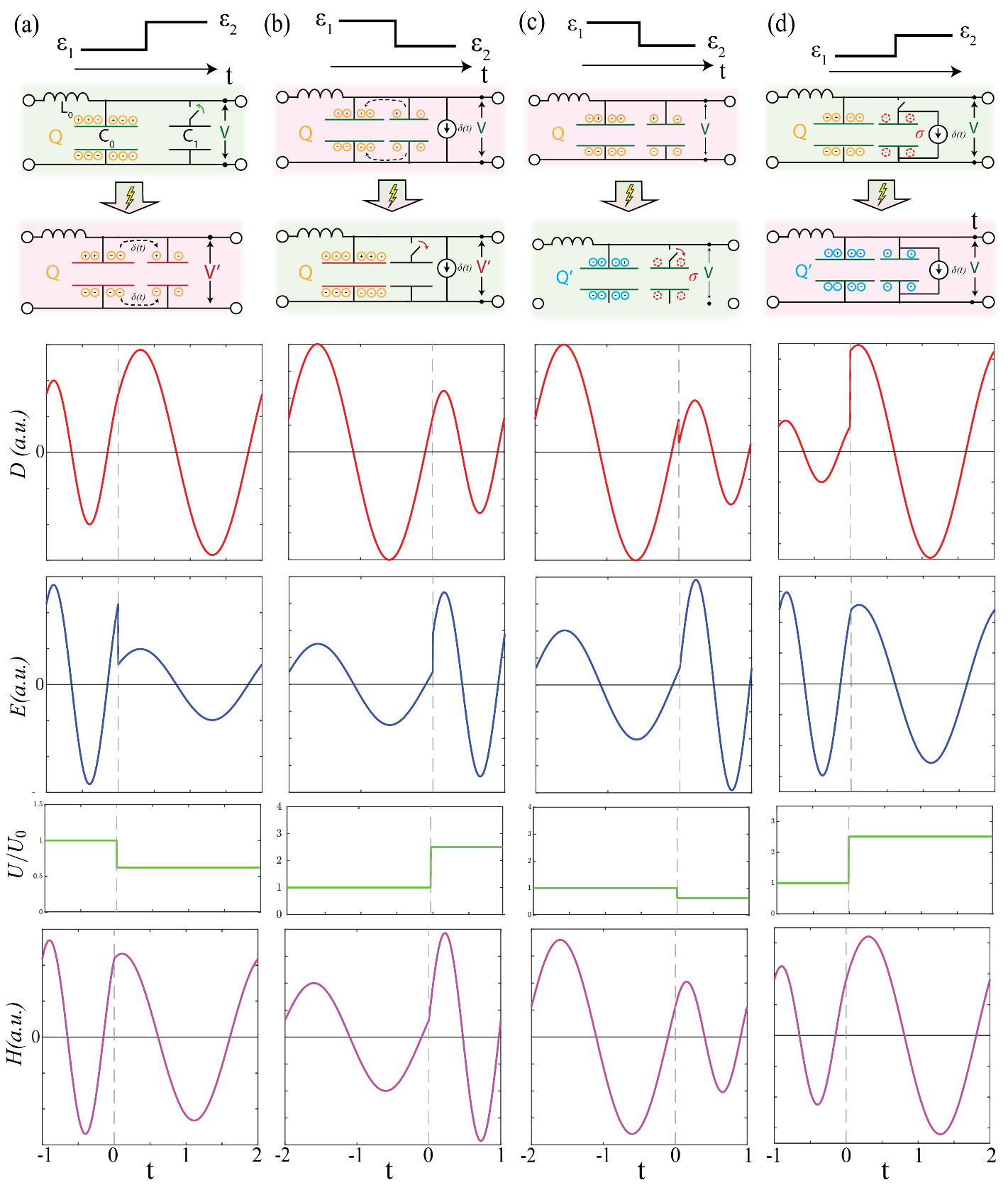
**

***Fig. S1:*** *Numerically computed (FDTD) field plots for (column a) a permittivity increase under charge conservation (displacement-field continuity), (column b) a permittivity reduction under charge conservation, (column c) a permittivity reduction under voltage continuity and (column d) a permittivity increase under voltage continuity.* *The plots in the four rows show respectively (top to bottom) the displacement field D, the electric field E, the energy density $U$ (normalized to the initial value $U_{0}$) and the magnetic field H, as a function of time.*

1. **Conditions for time-reflection cancellation and energy conservation**

The generalized time-reflection coefficient for a dielectric non-dispersive time interface is:

$R_{\sigma}=\frac{\varepsilon_{1}}{2\varepsilon_{2}}(1-\sqrt{\frac{\varepsilon_{2}}{\varepsilon_{1}}}+\bar{\sigma}_{se})$.

We shall assume that $\varepsilon_{2}<\varepsilon_{1}$, as that is the scenario where charges are typically dumped from the system. We note that $\bar{\sigma}_{se}=Z_{1}/Z_{2}-1$ perfectly cancels the reflection coefficient. What are the physical implications of this condition? Looking at the time-refraction coefficient for this case yields:

$$T_{\sigma}=\frac{\varepsilon_{1}}{2\varepsilon_{2}}[(1+\sqrt{\frac{\varepsilon_{2}}{\varepsilon_{1}})}+(\sqrt{\frac{\varepsilon_{2}}{\varepsilon_{1}}}-1)]=\sqrt{\frac{\varepsilon_{1}}{\varepsilon_{2}}}$$

Calculating the final electric energy, therefore, gives:

$$U/U_{0}=(\varepsilon_{2}T_{\sigma}^{2})/(\varepsilon_{1})=(\varepsilon_{2}\frac{\varepsilon_{1}}{\varepsilon_{2}})/(\varepsilon_{1})=1$$

This condition arises from the competition between the energy lost due to the loss of charge and the gain resulting by the permittivity reduction. Note that this scenario generally requires a gain mechanism which preserves or replaces a part of the charges which would otherwise be lost. This condition is possible because the energy due purely to the loss of charge (electric-field continuity) scales linearly with the permittivity variation ($U\sim\varepsilon|E|^{2}$) while that due to the variation of permittivity under charge conservation (displacement-field continuity) scales with the inverse of the permittivity ($U\sim|D|^{2}/\varepsilon$), such that a mixed boundary condition can exactly balance loss and gain. This can also be checked by verifying that Eq. for the electric field coefficients automatically satisfies the energy balances $D_{1}E_{1}=D_{2}E_{2}$, since the equivalent coefficient for the displacement field is obtained by multiplying $T_{\sigma}$ by $\varepsilon_{2}/\varepsilon_{1}$.

1. **Energy and momentum considerations for generalized non-dispersive time-interfaces**

It is meaningful to consider the change of electromagnetic energy and momentum upon such a generalized time-interface. Using Eqs. 7 in the main text, it is easy to find that the electric energy becomes:

$U/U_{0}=\frac{\varepsilon_{2}}{\varepsilon_{1}}(T_{\sigma}^{2}+R_{\sigma}^{2})=\frac{\varepsilon_{1}}{2\varepsilon_{2}}[(1+\bar{\sigma}_{se})^{2}+\frac{\varepsilon_{2}}{\varepsilon_{1}}]$,

whereas the momentum becomes:

$P/P_{0}=[1+\bar{\sigma}_{se}]$.

1. **Derivation of scattering coefficients in Drude time-interfaces upon flux conservation**

From Maxwell’s Equations before and after the time-interface, we have:

$$\begin{matrix} -i\omega\mu_{0}H_{y}=-ikE_{x} \\ -i\omega\varepsilon_{0}E_{x}=-ikH_{y}-J_{c} \\ -i\omega LJ_{c}=E_{x} \end{matrix}$$

This system hosts three modes: two ac modes with frequencies $\omega^{\pm}=\pm\sqrt{\omega_{0}^{2}+\omega_{p}^{2}}$, and eigenvectors:

$$\begin{matrix} E_{x}=\mu_{0}\frac{\omega}{k}H_{y} \\ J_{c}=\frac{i\mu_{0}}{kL}H_{y} \end{matrix}$$

and a DC ($\omega_{w}=0$) mode (sometimes called a “wiggler mode” in the plasma physics literature, see e.g. [2]), with eigenvector:

$$\begin{matrix} E_{x}=0 \\ J_{c}=-ikH_{y} \end{matrix}$$

Assuming that the initial wave is an ac mode with $H_{1}=H_{0}e^{i(kx-\omega_{1}^{+}t)}$ (we subsequently drop the subscripts x and y, since the problem is one-dimensional), where $\omega_{1}^{+}=\sqrt{\omega_{0}^{2}+\omega_{p,1}^{2}}$, and $\omega_{0}=c_{0}k$, the initial electric field and conduction current are given by

$$\begin{matrix} E_{1}=\mu_{0}\frac{\omega}{k}H_{1} \\ J_{c,1}=\frac{i\mu_{0}}{kL_{1}}H_{1} \end{matrix}$$

And the boundary conditions that encompass flux conservation read, upon some basic manipulation:

where , and are the magnetic-field scattering coefficients for the forward, backward and DC mode respectively. Solving this linear system yields the scattering coefficients in Eq. (11) of the main text.

1. **Scattering coefficients for the different Drude switching scenarios**


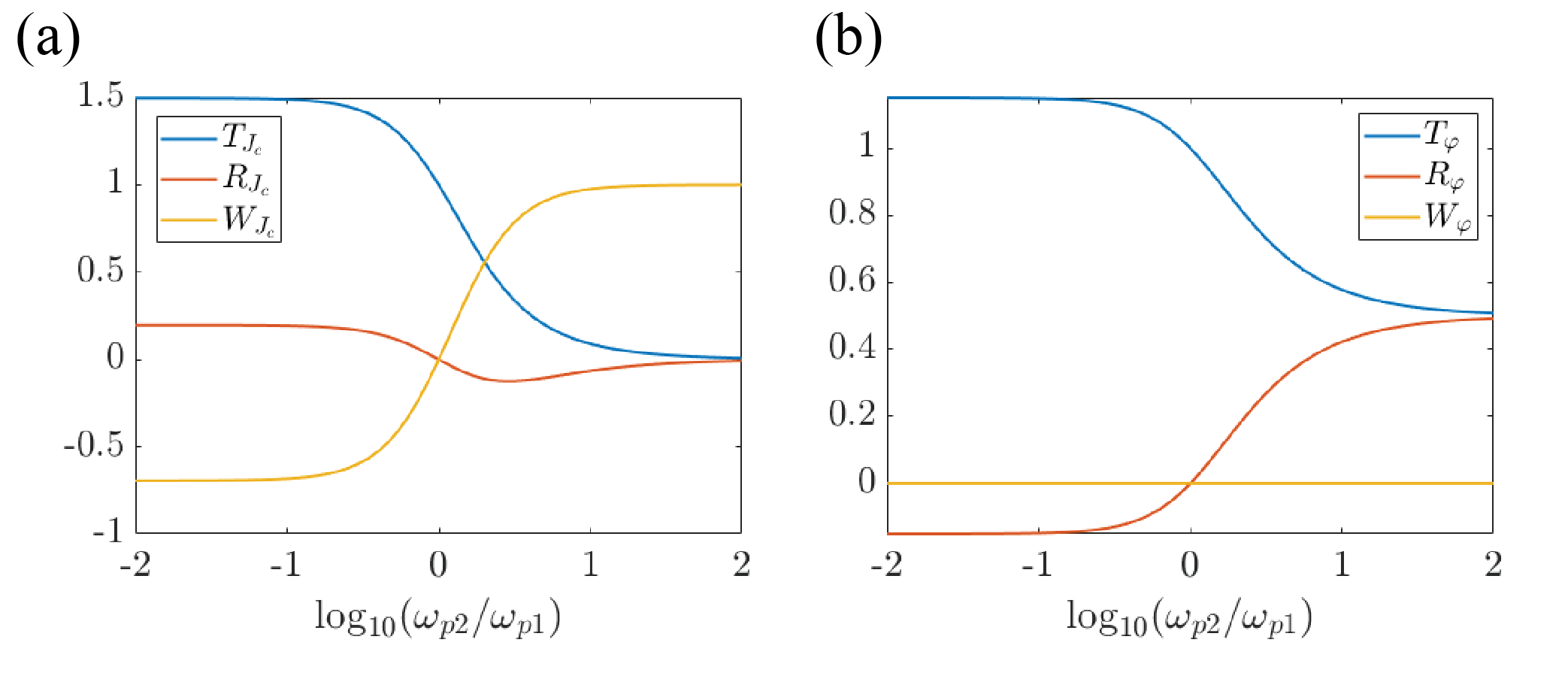


**Fig. S3:** *Magnetic-field* *scattering coefficients for the forward (T), backward (R) and DC/Wiggler (W) modes for the cases of (a) current continuity and (b) flux linkage continuity, as a function of the logarithm of the ratio between final and initial plasma frequency.*

1. **Numerical field plots for dispersive scenarios**

In the case of a plasma described by the Drude free-electron model, and assuming $\varepsilon_{\infty}=1$ for simplicity, Eq. becomes:

$$ikH_{y}(t)=\varepsilon_{0}\partial_{t}E_{x}(t)+J_{x}(t)$$

Following the inductor equivalence from the main text, we derive the Drude current as:

$$L(t)\partial_{t}J_{x}(t)=E_{x}(t)$$

which conserves $J$ across the temporal interface and applies to Figs. 3a-b and 3c-d of the main manuscript, or as:

$$\partial_{t}[L(t)J_{x}(t)]=E_{x}(t)$$

which conserves flux linkage (mechanical momentum) , in accordance with Figs. 3e-f and 3g-h in the main text. Similarly to Eq., in both cases the step-like change in $L(t)$ is described with the approximation of Eq. .

Fig. S4 shows the resulting finite-difference time-domain calculations of the conduction current $J_{c}$, flux linkage in the shunt branch , energy density $U$ (top plot) and magnetic field $H$ for the four cases discussed: (a) plasma frequency increase under current continuity, (b) plasma frequency decrease under current continuity, (c) plasma frequency decrease under flux linkage continuity and (d) plasma frequency increase under flux linkage continuity. Note the remnant DC offset in the magnetic field for the cases where the current is conserved (a,b), due to the excitation of the wiggler mode.

**
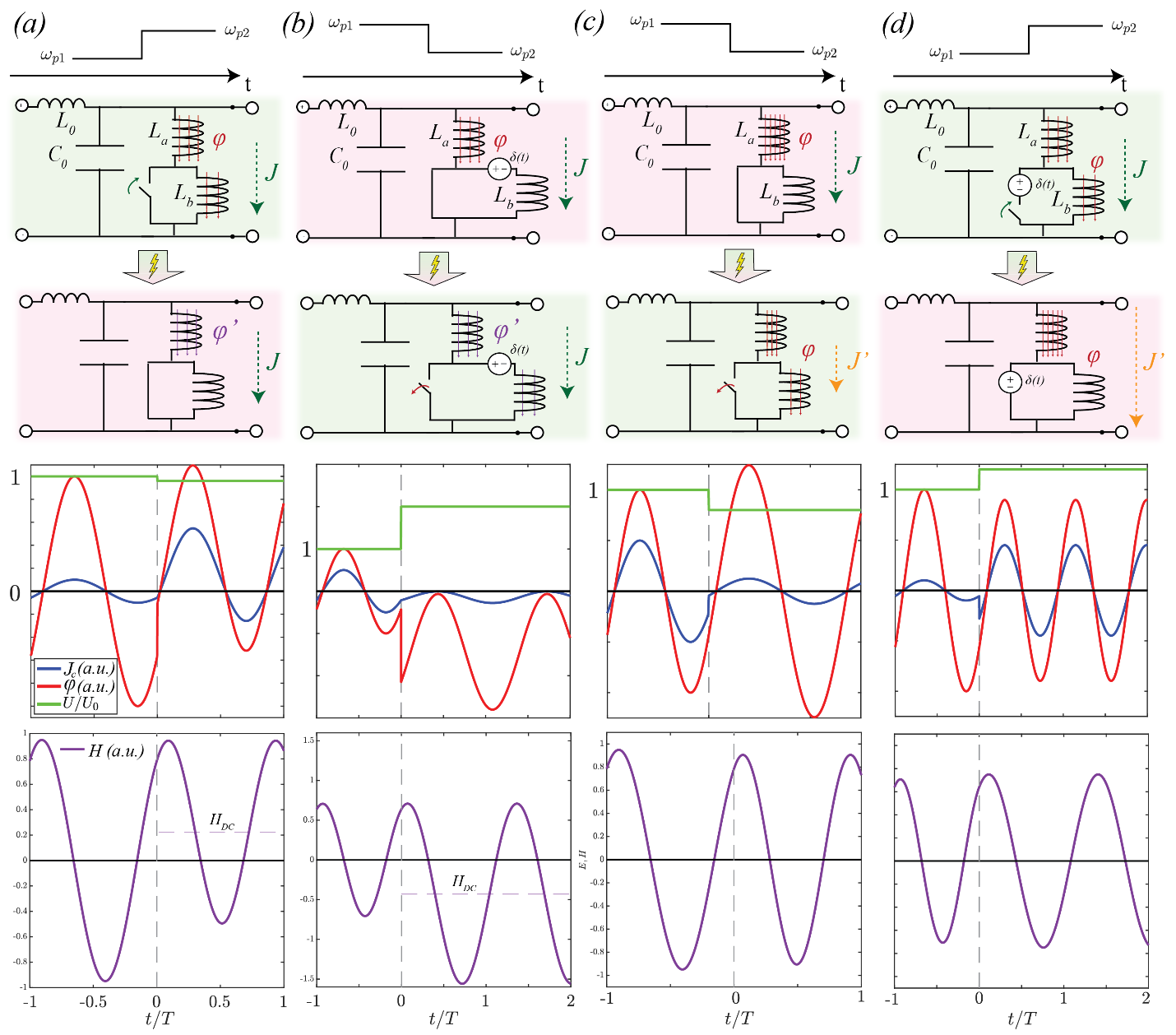
**

***Fig. S4:*** *Numerical field plots for the four cases considered in the main manuscript, showing current (blue), flux linkage (red), energy density (green) in the top plots, and the magnetic field purple) in the bottom plot.*

1. **Finite-difference-time-domain simulations in space (one dimension) and time**

*7a. Nondispersive scenarios*

All previous plots were restricted to plane wave propagation for a single $k$ component. Here we assume the initial conditions of a Gaussian pulse of envelope $e^{-[(t-\sqrt{\varepsilon_{1}}z/c)/\sigma]^{2}/2}$, and enforce the abrupt temporal transitions on $\varepsilon(t)$ in the (t,z) domain with our in-house FDTD solver. Fig. S5 shows the continuities/discontinuities on the normalized $E$ (upper row) and $E$ (lower row) fields corresponding to the four different scenarios (a-d) in Fig. S1 when $\sigma=T$, where $T$ is the period of the initial central frequency. This nondispersive nature allows for non-distorted temporally refracted and reflected pulses. Alternatively, in Fig. S6 we plot the analytic results in $(t,k)$ space, whose inverse Fourier transform identically recovers the colormaps of Fig. S5. Here $k_{0}$ denotes the initial central wavenumber.

***Fig. S5****: (t,z)-FDTD field plots for (column a) a permittivity increase under charge conservation (displacement-field continuity), (column b) a permittivity reduction under charge conservation, (column c) a permittivity reduction under voltage continuity and (column d) a permittivity increase under voltage continuity. The plots in the two rows show (top to bottom) the electric field E and the displacement field D, respectively, as a function of time (t) and space (z).*

***Fig. S6****: (t,k)-FDTD field plots for (column a) a permittivity increase under charge conservation (displacement-field continuity), (column b) a permittivity reduction under charge conservation, (column c) a permittivity reduction under voltage continuity and (column d) a permittivity increase under voltage continuity. The plots in the two rows show (top to bottom) the electric field E and the displacement field D, respectively, as a function of time (t) and wavevector (k).*

*7b. Dispersive scenarios*

The dispersive nature of the Drude model gives rise to a distorted pulse even before the time boundary. So let us first numerically solve the plane-wave problem from Fig. S4 in $\left( t,z \right)$ space, and choose Dirichlet spatial boundary conditions. As shown in Fig S7—representing the continuities/discontinuities on the normalized current $J$ (upper row) and flux linkage $\varphi$ (lower row) for the four different temporal transitions—, these create spatial reflections which, before the end of our simulation domain, do not reach the center region delimited by the two magenta horizontal lines, corresponding to one spatial period [$-\frac{\pi}{k},+\frac{\pi}{k}$]. Therefore these results are simply the $k$-phasor solution from Fig. S4 multiplied by $e^{-ikz}$, with the dashed white line at $z=\lambda/10$ representing the cut plotted in Fig. S4.

***Fig. S7****: (t,z)-FDTD plots for the four scenarios from Fig. S4: (column a) an increase in $\omega_{p}$ under current continuity, (column b) a reduction in $\omega_{p}$ under current continuity, (column c) a reduction in $\omega_{p}$ under flux linkage continuity, and (column d) an increase in $\omega_{p}$ under flux linkage continuity. The plots in the two rows show (top to bottom) the current field J and the flux linkage field $\varphi$, respectively, as a function of time (t) and space (z).*

Secondly, we consider that, before the temporal interface, our Drude medium is perturbed by the electromagnetic pulsed wave that results from an impressed current plate (infinitely extended in $x$ and $y$) of temporal envelope $e^{-\left[ \left( t-t_{0} \right)/\sigma\right]^{2}/2}$, with $t_{0}$ same instant prior to $t=0$. Fig. S8 represents the resulting temporally refracted and reflected $J$ and $\varphi$ pulses, where $\sigma$ has been doubled with respect to the nondispersive case. In addition, as expected, only the first two columns (a), (b) give rise to a (spatially varying) DC component, commensurate with current continuity.

***Fig. S8****: (t,z)-FDTD plots for the four scenarios from Fig. S4: (column a) an increase in $\omega_{p}$ under current continuity, (column b) a reduction in $\omega_{p}$ under current continuity, (column c) a reduction in $\omega_{p}$ under flux linkage continuity, and (column d) an increase in $\omega_{p}$ under flux linkage continuity. The plots in the two rows show (top to bottom) the current field J and the flux linkage field $\varphi$, respectively, as a function of time (t) and space (z).*

**8. Non-abrupt (tapered) temporal interfaces**

So far, we have demonstrated scenarios where the switching timescale is much shorter than the temporal cycle of the waves. Here we discuss scenarios where the permittivity varies continuously as a sigmoid function, varying the timescale of the soft step. Fig. S9 shows the dependence of the nondispersive scattering coefficients on the parameter $\alpha$ in Eq. , i.e. the rate of the temporal transition. In the adiabatic limit ($\alpha\ll T^{-1}$), there is no reflection ($R\to0$), which allows us to write the temporal refraction coefficients in compact form (black-colored asymptotes): under $D$-field continuity (solid lines), conservation of Minkowski momentum density $\mathbf{D}\times\mathbf{B}$ leads to $|T_{D}|\to(\varepsilon_{1}/\varepsilon_{2})^{3/4}$, whereas $E$-field continuity (dashed lines) conserves Abraham momentum density $\mathbf{E}\times\mathbf{H}$ and thus $|T_{E}|\to(\varepsilon_{1}/\varepsilon_{2})^{1/4}$ in the adiabatic limit [3].

***Fig. S9****: Forward (blue) and backward (red) scattering coefficients vs. rate $\alpha$ of temporal transition: (panel a) permittivity increase, (panel b) permittivity reduction. Solid (dashed) lines indicate D (E) continuity.*

Fig. S10 shows the corresponding results for the Drude scenario. Under continuity of the current $J$ (solid lines), we have $(\mathbf{E}\times\mathbf{H})_{t=0^{+}}=\frac{\varepsilon(\omega_{2})}{\varepsilon(\omega_{1})}(\mathbf{E}\times\mathbf{H})_{t=0-}$, which leads to $|T_{J}|\to[\frac{\varepsilon(\omega_{2})}{\varepsilon(\omega_{1})}]^{1/4}$ in the adiabatic limit. On the other hand, continuity of the magnetic flux (dashed lines) conserves $\mathbf{E}\times\mathbf{H}$ and thus in the adiabatic limit.

***Fig. S10****: Forward (blue), backward (red) and DC/Wiggler (yellow) scattering coefficients vs. rate $\alpha$ of temporal transition: (panel a) increase of plasma frequency, (panel b) reduction of plasma frequency. Solid and dashed lines indicate continuity of $J$and , respectively.*

**References**

1. A. Taflove, S. C. Hagness, *Computational Electrodynamics: The Finite-Difference Time-Domain Method*, Artech House, Boston, MA 2005.
2. Kalluri, D. K. (2018). *Electromagnetics of time varying complex media: frequency and polarization transformer*. CRC Press.
3. E. Galiffi, S. Yin and A. Alú, “Tapered photonic switching”, *Nanophotonics* **11** (16)*,* 2022.
